# Variability of Clinical Presentation in Patients Heterozygous for the F508del Cystic Fibrosis Variant: A Series of Three Cases and a Review of the Literature

**DOI:** 10.7759/cureus.40185

**Published:** 2023-06-09

**Authors:** Caitlin M Raymond, Simon P Gaul, Song Han, Gengming Huang, Jianli Dong

**Affiliations:** 1 Pathology, University of Texas Medical Branch, Galveston, USA; 2 Medicine, University of Texas Medical Branch, John Sealy School of Medicine, Galveston, USA

**Keywords:** heterozygous f508del, cftr mutation, cystic fibrosis carrier, atypical cystic fibrosis, cystic fibrosis (cf), cystic fibrosis transmembrane conductance regulator

## Abstract

Cystic fibrosis (CF) is a genetic disease that affects the lung, pancreas, and other organs caused by the presence of biallelic CF-causing variants in the cystic fibrosis conductance regular gene (CFTR). CFTR variants can also be found in CFTR-related disorders (CFTR-RD), which present milder symptoms. Increasing access to next-generation sequencing has demonstrated that both CF and CFTR-RD have a broader array of genotypes than formerly thought. Here we present three patients who carry the most common CFTR pathogenic variant - F508del - but express a wide array of phenotypes. These cases open discussion on the role of concurrent variants in CFTR, the importance of early diagnosis and treatment, and the contribution of lifestyle factors in CF and CFTR-RD presentation.

## Introduction

One of the most common life-shortening autosomal recessive diseases, cystic fibrosis (CF; OMIM 219700), affects approximately 40,000 individuals in the US and occurs in individuals of all ethnicities [1, 2]. Those affected experience a range of symptoms affecting multiple organs, including the lung, pancreas, liver, sinus, and the male reproductive tract. A diagnosis of CF has drastic implications for both the patient and their family. Only a few decades ago, patients with CF passed away from respiratory failure in their late adolescence or early twenties; however, extensive research and advances in treatment have expanded the expected lifespan of a patient with CF into their mid to late forties, with some patients living into their fifties and beyond [1, 2].

CF is caused by variants in the cystic fibrosis transmembrane conductance regulator gene (CFTR; OMIM 602421). The CFTR gene and the most common variant, c.1521_1523del (p.Phe508del; legacy: F508del or Phe508del), were first discovered in 1989 (OMIM 602421). In the more than 30 years since, over 2,000 additional variants in CFTR have been discovered [1, 2], with various molecular mechanisms ranging from defective protein synthesis, such as premature termination of messenger RNA translation, to errors in protein trafficking to the endoplasmic reticulum or cell membrane, such as protein misfolding. CFTR genetic variants are thus classified into six classes (I-VI) based on their molecular mechanism and ultimate impact on CFTR functionality, with the phenotypic severity of CF correlating with the amount of CFTR function at the cell membrane. Class IV, V, and VI variants are typically associated with milder disease, and class I, II, and III variants are associated with more severe disease. However, it is important to note many variants exhibit characteristics of more than one variant class. The phenotypic correlation of variants is equally complex. Phenotypic expressivity can be modulated by co-existing variants in the same or alternate CFTR allele, variants in the genetic background of the individual, and lifestyle factors such as tobacco and alcohol use [1, 2].

Over the past decades, there has been increasing recognition of CFTR-related disorders (CFTR-RD) [3, 4]. Currently, a diagnosis of CFTR-RD is made when a clinical presentation is associated with CFTR dysfunction but does not fulfill the diagnostic criteria for CF [3, 4]. In practice, patients with CFTR-RD often present with milder CF phenotypes in one or multiple organ systems with a normal or borderline sweat chloride or other CFTR function test and carry two CFTR variants. CFTR-RD can present with symptoms such as chronic sinusitis, nasal polyposis, focal biliary cirrhosis with portal hypertension, recurrent or chronic pancreatitis, or male infertility [3, 4]. Some organs may be more involved in CFTR-RDs due to differential splicing in tissues [5, 6]. Indeed, two previously documented clinical syndromes have been recognized to be associated with variants in CFTR: up to 20% of individuals with recurrent idiopathic pancreatitis have been found to carry biallelic CFTR variants [7], and up to 50% of patients affected with infertility secondary to congenital absence of the vas deferens (CAVD) carry biallelic CFTR variants [8, 9].

Heterozygous carriers of a CFTR variant may be more at risk of developing a CFTR-associated disorder, including bronchiectasis, allergic bronchopulmonary aspergillosis, asthma, chronic rhinosinusitis, likely in the presence of variants of other modifier genes or external factors [10]. Two studies have documented successful elexacaftor/tezacaftor/Ivacaftor treatment of patients with advanced lung disease and an F508del/unknown genotype [11, 12].

## Case presentation

Case one

A five-year-old Hispanic male was evaluated by otolaryngology for complaints of chronic congestion, snoring, and cough. His past medical history at that time was significant for respiratory syncytial virus (RSV) and pneumonia as a newborn, asthma, and allergic rhinitis. He had a negative CF newborn bloodspot screening result. Family history was negative for CF. At the initial visit, he was diagnosed with nasal turbinate hypertrophy and started on medical management consisting of nasal steroids, antihistamine, and montelukast; yet his symptoms failed to improve. A computerized tomography (CT) scan of the sinuses was ordered, which demonstrated severe nasal polyposis leading to obstruction and chronic ethmoid and maxillary sinusitis with bony degeneration.

He underwent bilateral ethmoidectomy and maxillary antrostomy with tissue removal. His breathing improved after his initial surgery, but several follow-up surgeries for nasal adhesions were necessitated. Currently, the patient's respiratory symptoms are well controlled on Ritchie's Nebulizer (prednisone + mupirocin).

Next-generation sequencing (NGS) of CFTR protein coding regions and intron/exon boundaries using TruSight Cystic Fibrosis assay (Illumina, San Diego) revealed he was heterozygous for F508del (NM_000492.3(CFTR):c.1521_1523del (p.Phe508del)), D1152H (NM_000492.4(CFTR):c.3454G>C (p.Asp1152His)), and V470M (NM_000492.4(CFTR):c.1408G>A (p.Val470Met)). The clinical significance of these variants is summarized in Table 1. Parental samples were not available to determine the cis vs. trans configuration of these variants. A sweat chloride test was not performed, but a subsequent fecal elastase was normal.

**Table 1:** A summary of CFTR NGS results from the three cases presented in this series The variants discovered in each case are listed with their CFTR2 and ClinVar designations. CF: cystic fibrosis, MVCC: variant of varying clinical consequence, VUCS: variant of varying clinical significance, VUS: variant of undetermined significance, COPD - chronic obstructive pulmonary disease

	Variant	CFTR2 Designation	ClinVar Designation	Presentation
Case 1	F508del	CF causing	Pathogenic	Severe nasal polyposis
	D1152H	MVCC	VUS	
	V470M	Non-CF causing	Benign	
Case 2	F508del	CF causing	Pathogenic	Cystic fibrosis with pancreatic insufficiency and bronchiectasis
	R74W	MVCC	VUS	
	V201M	VUCS	VUS	
	D1270N	MVCC	VUS	
	V470M	Non-CF causing	Benign	
Case 3	F508del	CF causing	Pathogenic	Chronic pancreatitis and COPD
	V470M	Non-CF causing	Benign	

Case two

A 38-year-old Caucasian male with a past medical history of CF diagnosed at age 12, complicated by bronchiectasis and pancreatic insufficiency, presented to the pulmonary clinic for management of chronic dyspnea. The patient had previously been treated for cystic fibrosis with Serevent, albuterol, inhalational tobramycin, and pancreatic enzymes, but he self-discontinued his medications and was lost to follow-up for some time. Upon representing, he reported chronic dyspnea at rest on beclomethasone and albuterol inhalers, with four to five exacerbations per year that manifested as sinusitis and migrated into his chest. Family history was positive for CF in the paternal grandfather.

CFTR NGS test revealed heterozygosity for F508del, R74W )NM_000492.4(CFTR):c.220C>T (p.Arg74Trp)), V201M (NM_000492.4(CFTR):c.601G>A (p.Val201Met)), V470M, and D1270N (NM_000492.4(CFTR):c.3808G>A (p.Asp1270Asn)) (Table 1). His clinical team noted his CF presentation was "mild" and he was started on dornase alfa, albuterol nebulizer, hypertonic 7% saline nebulizer, pancreatic enzymes, and a beclomethasone inhaler. At the following visit, he reported a complete resolution of his dyspnea on the new medical regimen and improvement in his functional capacity.

Case three

A 62-year-old Caucasian male presented with a productive cough and involuntary weight loss of one hundred pounds over the past year. He was noted to have a past medical history of alcohol use at 24 beers per week until age 35 when he was diagnosed with chronic pancreatitis. He was a former smoker with 30 pack per year history. He had a prior history of tonsillar squamous cell carcinoma, status post (s/p) surgical resection, and radiation treatment at age 56.

Two months prior to presentation, he was noted to have bilateral opacities on chest CT with a tree-in-bud appearance and bronchial wall thickening. Polymerase chain reaction (PCR) for mycobacterium tuberculosis was negative. Pulmonary function tests demonstrated an obstructive pattern, and the patient was diagnosed with chronic obstructive pulmonary disease (COPD).

At the time of presentation, the patient was noted to have acute hypoxic hypercapnic respiratory failure and, during his admission, was supported in the intensive care unit (ICU). A nasogastric tube was placed for feeding; however, the patient continued to lose weight and deteriorate. Noting the presence of chronic pancreatitis, weight loss despite feeding, and chronic obstructive pulmonary disease (COPD), the medical team ordered CFTR genetic test just before the patient passed away. The patient was posthumously found to be a heterozygous carrier of CFTR F508del and V470M variants by NGS sequencing, but no other variants, including deletions and duplications involving CFTR, were found on whole exome sequencing. Separate CFTR deletion/duplication analysis was not performed. Sweat chloride test and other CFTR functional tests were not performed.

## Discussion

CF and CFTR-RD have complex genotype-phenotype correlations due to variable expressivity and reduced penetrance of CFTR variants, which is currently thought to involve a number of factors both genetic and environmental. We present three cases that all carry one copy of the F508del CFTR variant but have drastically different outcomes exemplifying genotypic and phenotypic variability associated with this common CFTR variant. ClinVar and CFTR2 databases were used to facilitate the interpretation of sequence variants in these cases. ClinVar, a public archive of the relationships among human variations and phenotypes, is maintained by the National Institutes of Health. Variants are listed in ClinVar on a five-point scale according to American College of Medical Genetics and Genomics (ACMG) variant classification recommendations [13]: pathogenic, likely pathogenic, variant of undetermined significance (VUS), likely benign, and benign. In contrast, The Clinical and Functional Translation of CFTR (CFTR2) project, managed by Johns Hopkins University, focuses specifically on CFTR and its variants. In the CFTR2 database, variants are defined as: a CF-causing variant; a non-CF-causing variant; a variant of varying clinical consequence (MVCC); or a variant of unknown clinical significance (VUCS). Notably, a non-CF-causing variant may be associated with CFTR-RD or no disease, and MVCC may result in CF, CFTR-RD, or no disease when they are in trans with another CF-causing variant.

All three patients carry a pathogenic/CF-causing F508del variant. F508del is a deletion of three nucleic acids in the CFTR gene (NM_000492.3: c.1521_1523del), that leads to the loss of a phenylalanine at position 508 in the CFTR protein. This results in misfolding and degradation of the protein and a reduced amount of CFTR function at the cell membrane. It is a class II variant, meaning it leads to improper processing of the protein and trafficking to the cell membrane (Table 2). Research has also demonstrated that the F508del variant exhibits abnormal channel gating (characteristic of class III variants), and reduced channel stability (characteristic of class VI variants) [1, 2].

**Table 2:** The classes of CFTR variants The six classes of CFTR variants are listed, with an example variant from that class, and the molecular mechanism of action. Table credit: Caitlin Raymond, author. CFTR - cystic fibrosis conductance regular gene,

Variant Class	Variant Example	Mechanism of action
I	G542X	Absent of functional CFTR protein
II	F508del	Improper processing or transportation to cell membrane
III	G551D	Loss of CFTR chloride channel activation
IV	R117H	Decreased channel conductance
V	5T	CFTR protein is normally functioning, but there are reduced amounts at the cell membrane due to promoter or splicing variants (also includes abnormal trafficking of normally functioning CFTR protein)
VI	4326delTC	Decreased stability of normally functioning CFTR protein

In this study, case one carries F508del, D1152H, and V470M (Table 1). Parental samples in our cases are not available to determine cis vs. trans configuration of the variants. Both F508del and D1152H are listed as pathogenic in the ClinVar database, with F508del characterized as CF-causing and D1152H listed as an MVCC in the CFTR2 database. D1152H has been found to affect the anion selectivity of the CFTR protein, which can be rescued by ivacaftor [14]. F508del and D1152H have been identified as compound heterozygous in 43 patients carrying both variants [15]. Compound heterozygous for D1152H and F508del has been associated mostly with mild CF with a wide range of phenotypes, including the nasal polyposis seen in this study [15]. D1152H is one of the variants frequently encountered in patients with CFTR-RD [4]. Variant V470M has been found to affect protein maturity [16] but is largely considered benign on its own, acting mainly to affect the penetrance of other variants [16, 17].

In case two, F508del is found together with four variants, all of which are designated as VUS or Benign in ClinVar, and with designations ranging from MVCC to VUCS to Non-CF causing in CFTR2 (Table 1). Three of the variants in case two, R74W, V201M, and D1270N were previously found to be in cis as a complex allele p.(R74W;V201M;D1270N). This complex variant is associated with various clinical features, including CF and CFTR-RD, and is frequently found in patients with CFTR-RD [4, 18]. Notably, this patient is the only one in this case series to be diagnosed with CF, which was specified to be "mild" in his clinical notes. Details on the diagnostic methods when the patient was in childhood were unknown.

Finally, case three had arguably the worst outcome, with the most apparently benign genotype (Table 1); yet this patient presented CF symptoms of pancreatic insufficiency and respiratory failure. Unfortunately, the patient's risk for CF was not known until after his death, and no CFTR functional assays were performed. Whole exome sequencing of the patient's genome was notable for the absence of CFTR gene copy number variants and variants in genes with known modifier potential, suggesting that haploinsufficiency would have arisen from the F508del variant alone. This patient's history of alcohol and cigarette use are important variables to consider. The presence of the F508del variant likely led to a reduction in CFTR function at the cell membrane, which, combined with the additional insults of alcohol and cigarette use, could have produced haploinsufficiency of the CFTR gene and the development of a CF phenotype.

These lifestyle factors alone are sufficient to cause issues like chronic pancreatitis and COPD and may have contributed to the delayed recognition of possible CFTR-associated phenotypes. However, the question is the degree to which the patient's F508del variant predisposed him to develop a CFTR-associated phenotype. It is widely accepted that the severity of CFTR-associated phenotype correlates with the amount of CFTR protein function at the cell membrane, such that variants with reduced function of CFTR produce a milder phenotype than variants with absence of CFTR [3, 4]. Moreover, it has been previously established that patients with only one known variant - F508del - can present with a CFTR-associated phenotype as symptomatic heterozygous [10].

Early diagnosis and treatment of CF have been well established to lead to better patient outcomes through aggressive supportive treatment, including treatment of infection, anti-inflammatory treatment, particularly with macrolide antibiotics, airway clearance therapy, including treatment with inhaled hyperosmolar agents, and nutritional support. Recently, the treatment of CF has undergone a revolution with the development of CFTR-modulating therapy that targets the underlying characteristics of certain CFTR variants. For example, combination therapy with ivacaftor, which increases the conductance of variant CFTR channels, as well as tezacaftor and elexacaftor, which stabilize misfolded F508del variant protein, have shown clinical efficacy in patients with advanced lung disease who are heterozygous for F508del [11, 12].

Since the discovery of a genetic basis for CF, various programs have been initiated to increase screening in the population. Currently, in the United States, most newborns receive genetic screening, including CF at birth. Furthermore, many individuals are assessed for CFTR variants through pre-conception or pre-marital screening, particularly in populations that have a high carrier rate. Historically, most screening for CFTR variants has occurred only at birth or during reproductive counseling, but this is beginning to change, and at least in patients with a CF or CFTR-RD phenotype, there is a recent effort to improve genetic tests for CFTR variants. For example, in 2017, the European Respiratory Society released new guidelines recommending routine screening for CFTR variants in certain patients with bronchiectasis [19].

While our knowledge of the wide array of clinical presentations in CF and CFTR-RD has grown in the past decades, researchers have also uncovered evidence that CFTR variants affect innate and adaptive immunity [20]. CF is well-known to feature inflammation and recurrent infection in the lungs, and recent evidence suggests that CFTR variants affect the neutrophil function and enhance the release of inflammatory cytokines [21]. In the same vein, researchers are finding that CF patients are more prone to autoimmune diseases [20]. In particular, CF patients have been found to have celiac disease with a prevalence over twice as high as the global population [20].

## Conclusions

CFTR variants are associated with variable disease expressivity and reduced penetrance. As access to molecular diagnostics expands, our understanding of the complexity of CF and CFTR-RD will deepen, opening the possibility for earlier diagnosis and treatment in patients with CFTR variants - whether alone or in trans to other variants - who are at greater risk of developing a CFTR-related phenotype.
